# Effectiveness of GnRH Antagonist in the Management of Subfertile Couples Undergoing Controlled Ovarian Stimulation and Intrauterine Insemination: A Meta-Analysis

**DOI:** 10.1371/journal.pone.0109133

**Published:** 2014-10-09

**Authors:** Shan Luo, Shangwei Li, Song Jin, Ya Li, Yaoyao Zhang

**Affiliations:** Division of Reproductive Medical Center, West China Second University Hospital of Sichuan University, Chengdu, Sichuan, China; University of Aberdeen, United Kingdom

## Abstract

**Background:**

Recent studies have indicated the use of gonadotropin-releasing hormone antagonists (GnRH-ant) as an adjuvant treatment to prevent premature luteinization (PL) and improve the clinical outcomes in patients undergoing controlled ovarian stimulation (COS) with intrauterine insemination (IUI). However, the results of these studies are conflicting.

**Methods:**

We conducted a systematic review and meta-analysis of randomized trials aiming to compare the clinical efficacy of GnRH-ant in COS/IUI cycles. Twelve studies were identified that met inclusion criteria and comprised 2,577 cycles assigned to COS/IUI combined GnRH-ant or COS/IUI alone.

**Results:**

Meta-analysis results suggested that GnRH-ant can significantly increase the clinical pregnancy rate (CPR) (OR = 1.42; 95% CI, 1.13–1.78) and decrease the PL rate (OR = 0.22, 95% CI, 0.16–0.30) in COS/IUI cycles. Subgroup analysis results suggested statistically significant improvement in the CPR in non-PCOS patients (OR = 1.54; 95% CI, 1.03–2.31) but not in the PCOS population (OR = 1.65; 95% CI, 0.93–2.94) and multiple mature follicle cycles (OR = 1.87; 95% CI, 0.27–12.66). There were no difference in the miscarriage and multiple pregnancy rates between the groups.

**Conclusion:**

This meta-analysis suggested that GnRH-ant can reduce the incidence of PL and increase the CPR when used in COS/IUI cycles, and it was especially useful for non-PCOS patients. However, evidence to support its use in PCOS patients is still insufficient

## Introduction

Intrauterine insemination (IUI) combined with controlled ovarian stimulation (COS) with low-dose recombinant FSH (rFSH) is widely used in the treatment of unexplained, endometriosis or male factor infertility because of the higher pregnancy rates associated with this approach compared to IUI cycles without COS or those with clomiphene citrate stimulation [1]–[4]. The underlying mechanism is based on the increase in the number of available ova at the site of fertilization by ensuring 2–3 dominant follicles [3]. However, the recruitment of multiple follicles following COS rapidly increases the serum estradiol (E2) levels, which may increase the risk of a premature surge of luteinizing hormone (LH) and lead to premature luteinization (PL) in some cycles. PL has been reported to be detrimental to oocyte quality, fertilization, and embryo implantation [5], [6]. Approximately 20% of COS/IUI cycles have been shown to undergo PL [7].

Gonadotropin-releasing hormone antagonists (GnRH-ant) have been successfully used in IVF cycles to prevent premature LH surge for many years [8]. Their suppressive effect on the secretion of gonadotropins from the pituitary is mediated immediately after administration. Many research groups have investigated the use of GnRH-ant in women undergoing COS/IUI treatment to determine its benefits in improving the reproductive outcomes. However, the results from these studies are conflicting. The aim of this meta-analysis was to review current studies in which GnRH-ant was used in COS/IUI cycles, and to evaluate its efficacy in terms of clinical outcome.

## Materials and Methods

The Medline, EMBASE, Cochrane Library databases, ClinicalTrials.gov and the World Health Organization International Trials Registry Platform search portal were searched with no time limit applied to any database. A combination of Medical Subject Headings (MeSH) and text key words were used to generate three subsets of citations: for studies of GnRH-ant (using the key words “gonadotropin-releasing hormone antagonist” or “Ganirelix” or “Cetrorelix” or “Cetrotide”), COS (“controlled ovarian stimulation” or “ovarian stimulation” or “gonadotropin” or “FSH”), and IUI (“intrauterine insemination”). These subsets were combined using “AND” to generate a subset of citations relevant to the research question. The last updated search was performed in November 2013. In addition, the citation lists of relevant publications, review articles, abstracts of scientific meetings and included studies were manually searched to identify other potentially eligible studies. Studies published in languages other than English were not considered for inclusion. The study selection was undertaken by two of the authors of this work (YL and YYZ).

### Study selection and data extraction

Criteria for inclusion in the analysis were established before the literature search. Inclusion criteria were as follows: (1) published studies, (2) enrolled study participants were subfertile and for whom COS/IUI treatment was indicated, (3) patients had been treated with GnRH-ant (GnRH-ant group) concurrently with COS/IUI and were compared to patients treated with COS/IUI alone (control group). Two reviewers (YL and YYZ) used these criteria to review each article identified independently.

A study was excluded if: (1) the trial was not a RCT, (2) the patients accepted other assisted reproductive techniques instead of IUI after COS, such as IVF-ET, (3) the report was repeated or included identical patients in two studies (only the most recent article was included).

### Data collection

Systematic review was performed in accordance with the Preferred Reporting Items for Systematic Reviews and Meta-Analyses (PRISMA) guidelines. Two reviewers (YL and YYZ) completed the data extraction and quality assessment independently. When necessary, the reviewers wrote to the authors to obtain extra information and raw data. Inconsistencies between reviewers' data were resolved through discussion until a consensus was achieved. Outcome data from each study were extracted in 2×2 tables by YL and YYZ.

### Main outcomes

The clinical pregnancy rate (CPR) was the primary outcome of interest, and the PL, miscarriage, multiple pregnancies, and ovarian hyperstimulation syndrome (OHSS) rates were used as secondary outcomes. The CPR and PL were calculated on the basis of the number of cycles randomized across all studies, even if some cycles were cancelled or dropped out after randomization. PL was defined as a premature rise in the LH combined with a rise in the serum progesterone level. However, the threshold values of premature LH and progesterone increases differed slightly across the reviewed studies, because there are no unified criteria for these values at present.

### Risk of bias in individual studies

To ascertain the validity of each study, the methodological quality of all the selected trials was analyzed on the basis of information reported in the original published papers. The study quality was scrutinized by checking the adequacy of randomization, allocation concealment, the use of blinding, the completeness of follow-up, and outcome reporting according to the Cochrane Collaboration's tool for assessing risk of bias [9]. When these details were unclear in the initial publication, we contacted the authors to provide further clarification.

### Statistical analysis

Dichotomous outcomes were expressed as an odds ratio (OR) with 95% confidence intervals (CI). Heterogeneity of treatment effects was evaluated statistically using the *I* ^2^ statistic to quantify heterogeneity across studies [10]. If *I* ^2^≤50%, the variation between the studies was considered to be homogenous, and the fixed effects model was adopted for analysis. If *I* ^2^≥50%, there was significant heterogeneity between studies, the random effects model was adopted for analysis. The causes of heterogeneity were explored by investigating the variations in features of the population, exposure, and study quality.

Subgroup analyses were performed according to the study population and number of mature follicles on the human chorionic gonadotrophin (hCG) day. Statistical analyses were carried out using RevMan 5.0 software (Cochrane Collaboration, Oxford, UK).

## Results

**Figure 1:**
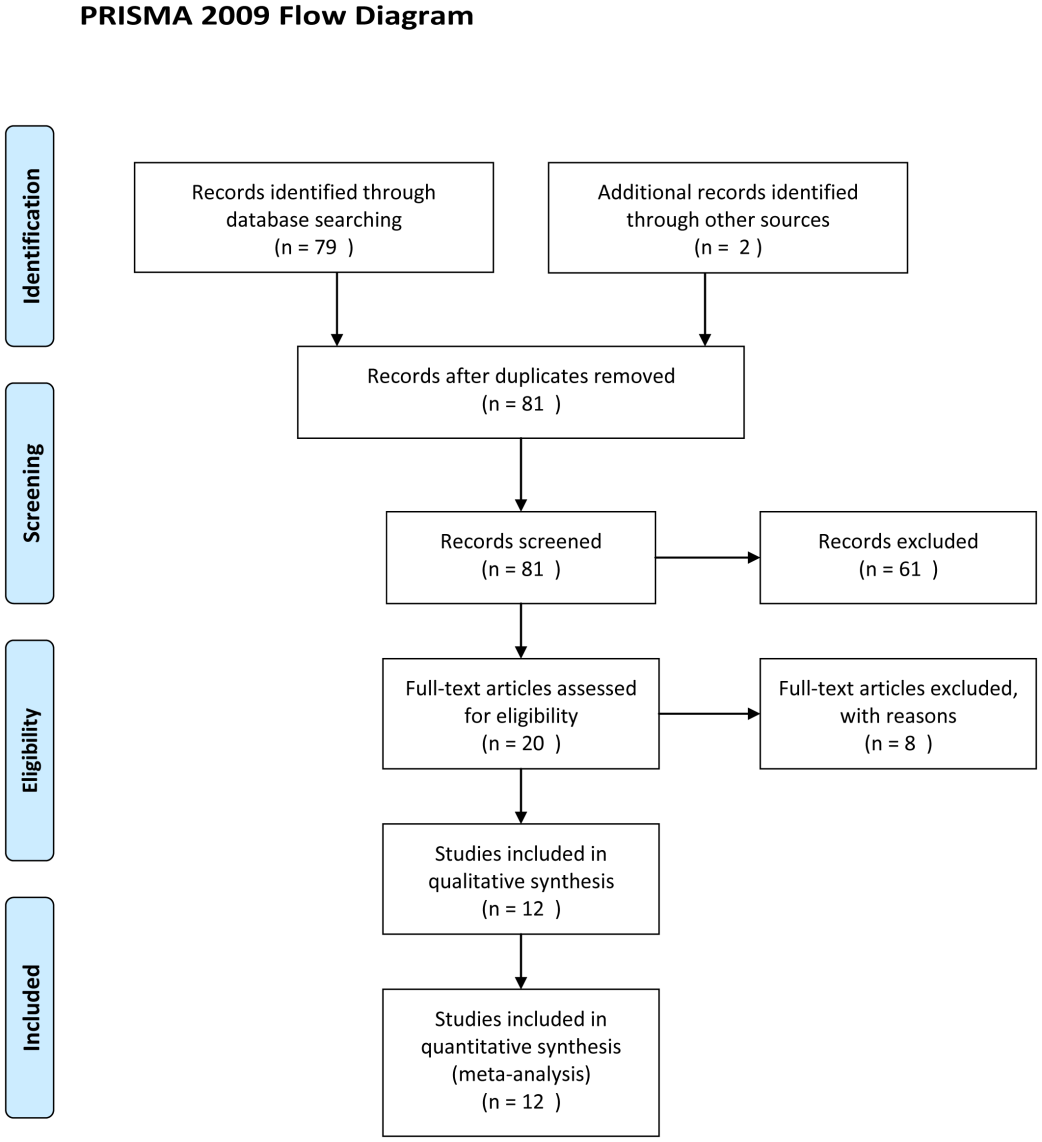
Flowchart of the study selection.

The literature search yielded 81 citations. Of these, 61 were excluded after reading the title and the abstract. Twenty full articles were retrieved for review. On examination of the manuscripts, eight studies were excluded [11]–[18]: three were not prospective randomized trials [11]–[13], two compared different gonadotropin doses [14], [15], one compared different doses of GnRH-ant [16], and the remaining two aimed to study the effectiveness of GnRH-ant in COS/IUI cycles to avoid IUI on weekends [17], [18]. The aims of the latter two studies were inconsistent with the aim of our meta-analysis, and postponing IUI to ensure that it is not carried out on weekends itself may make the physicians miss the best operation time and compromise the clinical outcome; therefore, these two studies were not included into our meta-analysis. A total of 12 randomized studies met the final including criteria [19]–[30]. Figure 1 illustrates a flowchart of the included studies.

Then, 1,256 cycles were assigned to the GnRH-ant group and 1,321 cycles to the control group. Ten studies used computer-generated randomization [19]–[24], [27]–[30] and two studies used randomization lists [25], [26]. Four studies were multicenter trials [20], [26], [27], [28]. Two studies used placebo and double-blinded methods [20], [26]. In eight studies, the participants were couples who had regular ovulation but with subfertility issues, including unexplained infertility, mild male factor infertility, and endometriosis (stages I–II) [20], [22]–[28]. In two studies, the participants were PCOS diagnosed on the basis of the Rotterdam consensus 2003 [29], [30]. In another two studies, women with all of the mentioned types of infertility were enrolled [19], [21]. The main demographic parameters between the treatment and control groups were comparable in the included studies. Eleven trials used recombinant FSH (rFSH) [19]–[21], [23]–[30], and one trial used letrozole + rFSH for the COS protocol [22]. Six studies used Ganirelix [24], [26]–[30], five studies used Cetrorelix [20]–[23], [25], and one study used Ovurelix [19]. The GnRH-ant protocols were similar in all studies, and had been applied from the late follicular phase. In all studies, hCG was administered when at least one leading follicle reached a diameter of ≥18 mm, and IUI was arranged 36–40 hours later. The main characteristics and the quality of the included studies are presented in Tables I, II, and III.

**Table 1:** Summary of study characteristics and clinical outcomes.

Study	Design	Quality features	Inclusion criteria	Participants	Intervention	Clinical outcomes
Kamath et al, 2013	RCT	Sample size calculation: no Adequate Randomization:yes Blinding: no	Infertility patients, including: mild male factor; unexplained infertility; minimal/mild endometriosis; and anovulation. Baseline characteristics were similar in both groups.	N = 141 GnRH-ant(n = 71) Age = 29.08±3.08 BMI = 24.1±1.2 Controls(n = 70) Age = 28.44±3.5 BMI = 23.5±2.3	Stimulation protocol: 75u/day of urinary gonadotrophins was given from day 3 for 3days, and adjusted later. Study group: Ovurelix was given when the lead follicle reached 14 mm till hCG day. Control:no	Clinical pregnancy rate Miscarriage rate Premature luteinization rate
Cantineau et al, 2011	RCT	Sample size calculation: yes Adequate Randomization:yes Blinding: yes	Infertility patients, including: mild male factor; unexplained infertility; minimal/mild endometriosis. Baseline characteristics were similar in both groups.	N = 233 GnRH-ant (n = 113) Age = 32.6±3.5 BMI = 23.1±3.3 Contros (n = 120) Age = 32.0±3.7 BMI = 23.6±3.9	Stimulation protocol: 75u/day rFSH was given from day3, and adjusted later. Study group:0.25 mg/day Cetrorelix was given when the lead follicle reached 14 mm till hCG day. Control: placebo	Live birth rate Clinical pregnancy rate Miscarriage rate Premature luteinization rate
Steward et al, 2011	RCT	Sample size calculation: yes Adequate Randomization:yes Blinding: no	Infertility patients, including: mild male factor; unexplained infertility; minimal/mild endometriosis; and anovulation. Baseline characteristics were similar in both groups.	N = 80 GnRH-ant (n = 40) Age:18–39 Contros (n = 40) Age:18–39	Stimulation protocol: 75–150u/day recombinant FSH (rFSH) was given from day2–4, and adjusted later. Study group: 0.25 mg/day Cetrorelix was given when the lead follicle reached 14 mm till hCG day. Control: no	Clinical pregnancy rate Miscarriage rate Premature luteinization rate OHSS rate
Stadtmauer et al., 2011	RCT	Sample size calculation: yes Adequate Randomization: yes Blinding: no	Infertility patients with PCOS. Baseline characteristics were similar in both groups.	N = 107 GnRH-ant (n = 54) Age = 30.8±3.9 BMI = 29.9±7.5 Contros (n = 53) Age = 29.9±4.2 BMI = 32.2±8.5	Stimulation protocol: 50–150u/day rFSH was given from day3, and adjusted later. Study group: 0.25 mg/day Ganirelix was given when the lead follicle reached 13 mm till hCG day. Control: no	Clinical pregnancy rate Miscarriage rate Premature luteinization rate
Ertunc et al, 2010	RCT	Sample size calculation: no Adequate Randomization: yes Blinding: no	Infertility patients with PCOS. Baseline characteristics were similar in both groups.	N = 226 GnRH-ant (n = 105) Age = 31.3±4.8 BMI = 26.1±3.6 Contros (n = 121) Age = 29.7±6.1 BMI = 24.9±4.3	Stimulation protocol: 50–150u/day rFSH was given from day3, and adjusted later. Study group: 0.25 mg/day Ganirelix was given when the lead follicle reached 13 mm till hCG day. Control: no	Clinical pregnancy rate Miscarriage rate Premature luteinization rate
Lee et al, 2008	RCT	Sample size calculation: no Adequate Randomization: yes Blinding: no	Infertility patients, including: mild male factor; unexplained infertility. Baseline characteristics were similar in both groups.	N = 61 GnRH-ant (n = 31) Age = 33.0±0.5 BMI = 20.7±0.9 Contros (n = 30) Age = 32.4±0.5 BMI = 20.5±0.6	Stimulation protocol:5 mg/day letrorole for 5 days, and 150u/alternate day rFSH was given later. Study group: 0.25 mg/day Cetrorelix was given when the lead follicle reached 14 mm till hCG day. Control: no	Clinical pregnancy rate Miscarriage rate Premature luteinization rate

**Table 2:** Summary of study characteristics and clinical outcomes.

Study	Design	Quality features	Inclusion criteria	Participants	Intervention	Clinical outcomes
Gomez et al, 2008	RCT	Sample size calculation: no Adequate Randomization:yes Blinding: no	183 infertility patients with regular menstrual cycles, normal uterine cavity, bilateral tubal patency, and normal endocrine function. Baseline characteristics were similar in both groups.	N = 367 GnRH-ant (n = 184) Age = 32.89±2.5 BMI:18–30 Contros (n = 183) Age = 32.05±3.9 BMI:18–30	Stimulation protocol: 75–150u/day rFSH was given from day3 or 4 for 5days, and adjusted later. Study group: 0.25 mg/day Cetrotide was given when the lead follicle reached 16 mm till hCG day. Control: no	Clinical pregnancy rate Multiple pregnancy rate OHSS rate
Allegra et al, 2007	RCT	Sample size calculation: yes Adequate Randomization:yes Blinding: no	Infertility patients, including: mild male factor; unexplained infertility; minimal/mild endometriosis, Baseline characteristics were similar in both groups.	N = 104 GnRH-ant (n = 52) Age = 33.0±4.0 BMI = 22.9±1,7 Contros (n = 52) Age = 32.5±3.6 BMI = 23.4±2.2	Stimulation protocol: 75–150u/day rFSH was given from day3, and adjusted later. Study group: 0.25 mg/day Ganirelix was given when the lead follicle reached 13 mm till hCG day. Control: no	Clinical pregnancy rate Miscarriage rate Premature luteinization rate OHSS rate
Crosignani et al. 2007	RCT	Sample size calculation: yes Adequate Randomization:yes Blinding: no	Infertility patients, including: mild male factor; unexplained infertility; minimal/mild endometriosis, Baseline characteristics were similar in both groups.	N = 299 GnRH-ant (n = 148) Age = 31.3±3.9 BMI = 22.5±3.1 Contros (n = 151) Age = 31.2±3.9 BMI = 22.6±3.0	Stimulation protocol: 50u/day rFSH was given from day3 till hCG day. Study group: 0.25 mg/day Ganirelix was given when the lead follicle reached 13–14 mm till hCG day. Control: no	Clinical pregnancy rate ongoing pregnancy rate Multiple pregnancy rate OHSS rate
Lambalk et al. 2006	RCT	Sample size calculation: yes Adequate Randomization: yes Blinding: yes	Infertility patients, including: mild male factor; unexplained infertility. Baseline characteristics were similar in both groups.	N = 204 GnRH-ant (n = 104) Age = 32.7±3.3 BMI = 22.9±3.0 Contros (n = 100) Age = 32.5±3.9 BMI = 23.3±3.1	Stimulation protocol: rFSH was given from day 2 or 3, and adjusted later. Study group:0.25 mg/day ganirelix was given when the lead follicle reached 14 mm till hCG day. Control: placebo	Clinical pregnancy rate Miscarriage rate Premature luteinization rate
Gomez et al, 2005	RCT	Sample size calculation: no Adequate Randomization:yes Blinding: no	40 infertility patients with regular menstrual cycles, normal uterine cavity, bilateral tubal patency, and normal endocrine function. Baseline characteristics were similar in both groups.	N = 82 GnRH-ant (n = 40) Age = 33.9±2.6 BMI:19–25 Contros (n = 42) Age = 32.05±3.3 BMI:19–25	Stimulation protocol: 100u/day recombinant FSH (rFSH) was given from day3–4 till hCG day. Study group: 0.25 mg/day Ganirelix was given when the lead follicle reached 16 mm till hCG day. Control: no	Clinical pregnancy rate Miscarriage rate Multiple pregnancy rate
Williams et al, 2004	RCT	Sample size calculation: yes Adequate Randomization:yes Blinding: assessor blind	54 infertility patients with regular menstrual cycles, normal uterine cavity, bilateral tubal patency, and normal endocrine function. Baseline characteristics were similar in both groups.	N = 118 GnRH-ant (n = 52) Age:18–39 BMI:18–35 Contros (n = 66) Age:18–39 BMI:18–35	Stimulation protocol: 2 ampules/day Follistim (rFSH) was given from day2 or 3 for 5days, and adjusted later. Study group: 0.25 mg/day Ganirelix was given on day 6 of Follistim treatment till hCG day. Control: no	Clinical pregnancy rate Multiple pregnancy rate

**Table 3:** Assessment of the risk of bias for included studies.

Study	Adequate randomization	Allocation concealment	Blinding of participants and personnel	Blinding of outcome assessment	Incomplete outcome data	Selective reporting
amath et al, 2013	Yes	Yes	No	No	No	Yes
Cantineau et al, 2011	Yes	Yes	Yes	Yes	No	No
Steward et al, 2011	Yes	No	No	No	Yes	Yes
Stadtmauer *et al*., 2011	Yes	Yes	No	No	No	No
Ertunc et al, 2010	Yes	Yes	No	No	No	Yes
Lee et al, 2008	Yes	Yes	No	Yes	No	Yes
Gomez et al, 2008	Yes	No	No	No	No	Yes
Allegra et al, 2007	Yes	Yes	No	No	No	Yes
Crosignani et al. 2007	Yes	Yes	No	No	No	Yes
Lambalk et al. 2006	Yes	No	Yes	Yes	No	Yes
Gomez et al, 2004	Yes	No	No	No	No	Yes
Williams et al, 2004	Yes	Yes	No	Yes	No	Yes

### Pregnancy outcomes

**Figure 2:**
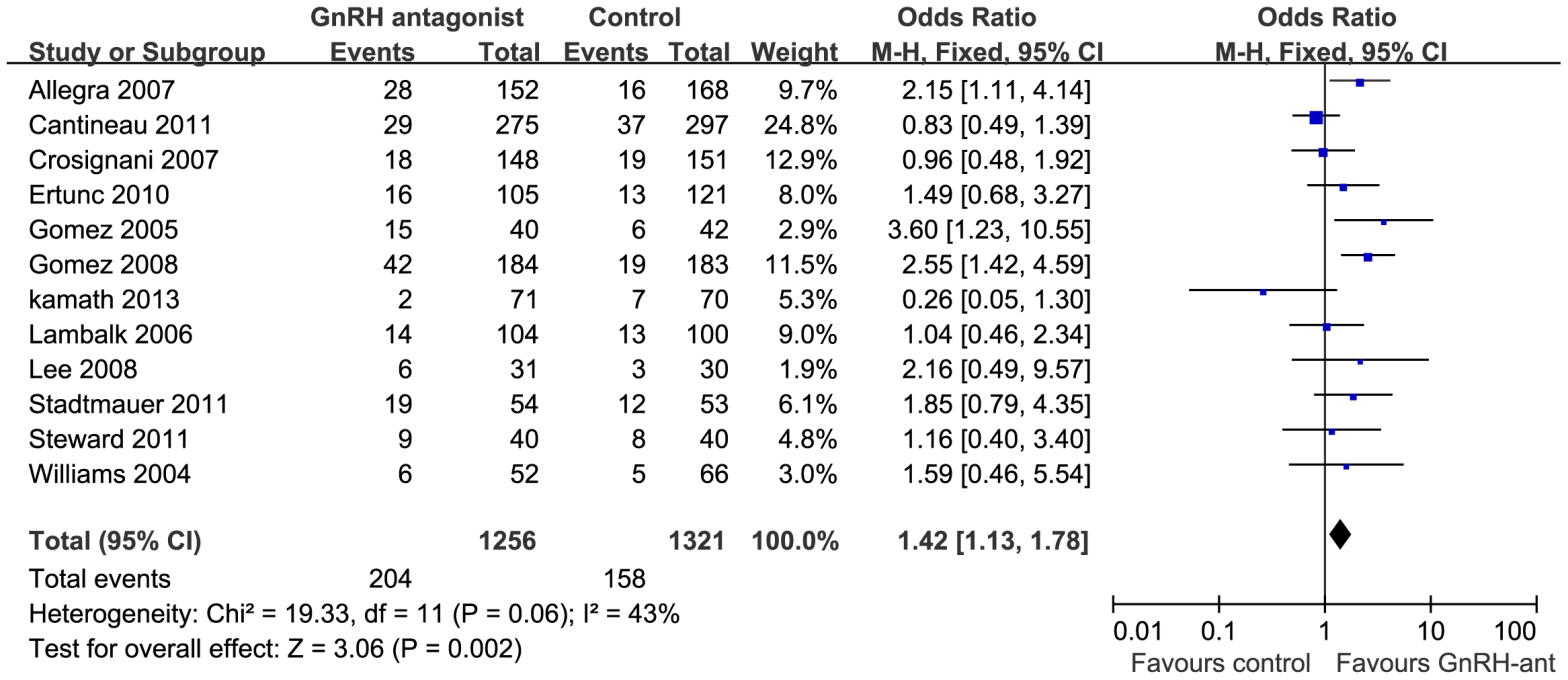
Forest plot of odds ratios (ORs) and 95% confidence interval (CI) of pooled trials comparing GnRH antagonist and control for clinical pregnancy rate.

All studies reported the CPR. In the heterogeneity analysis, there was no inter-study heterogeneity for CPR (I^2^ = 43%, p = 0.06). Therefore, we chose the fixed effects model to synthesize data. The results of the meta-analysis showed a 42% increase in CPR for GnRH-ant treatment compared with the control group (OR = 1.42; 95% CI, 1.13–1.78; Figure 2).

Eight studies compared multiple pregnancies [20], [21], [23]–[27], [29] and nine studies compared the miscarriage rates [20], [21], [23]–[27], [29], [30], and no statistically significant differences were found between the study and control groups in both cases (OR = 1.12; 95% CI, 0.51–2.47 and OR = 1.07; 95% CI, 0.55–2.07 respectively).

**Figure 3:**
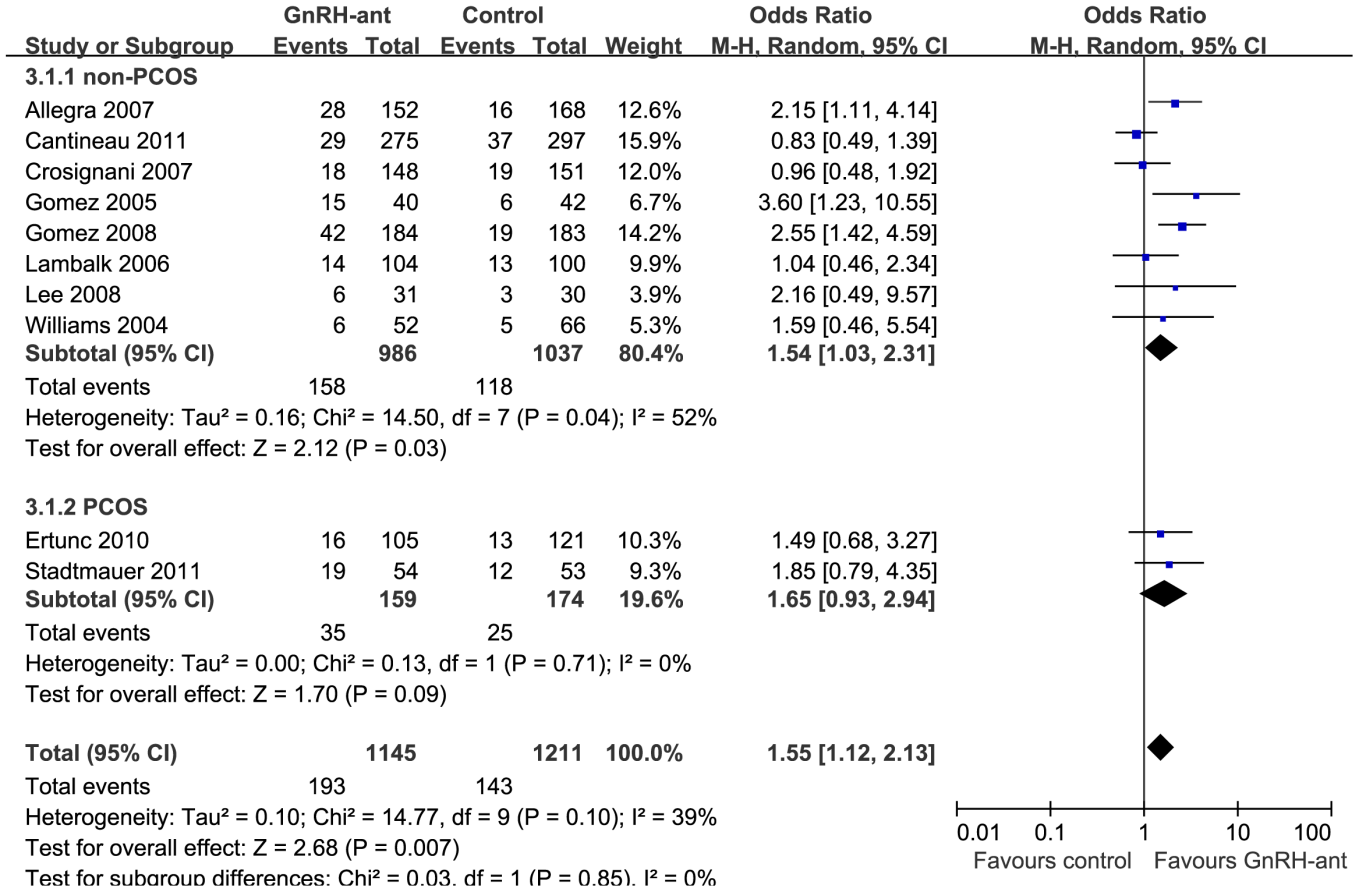
Subgroup analyses for the clinical pregnancy rate after GnRH antagonist administration vs. control for non-PCOS and PCOS patients.

For a further understanding of the effectiveness of GnRH-ant in clinical pregnancy outcomes in COS/IUI, subgroup analyses according to the reason for infertility and mature follicle number were performed. Eight studies included infertile women with normal ovulation, including cases of unexplained subfertility, mild male factor, or stage I–II endometriosis [20], [22]–[28], two studies included patients with PCOS [29], [30] who were infertile due to anovulation, and two studies included all the above cases of infertility [19], [21]. Meta-analysis of studies for non-PCOS patients showed a 54% increase in the CPR in the GnRH-ant group compared to the control group (OR = 1.54; 95% CI, 1.03–2.31; Figure 3). However, pooling data of patients with PCOS showed no difference between the GnRH-ant and the control group (OR = 1.65; 95% CI, 0.93–2.94; Figure 3).

Two studies reported the CPRs according to the number of mature follicles [20], [28]. Synthesis of single mature follicle cycles showed no significant difference between the two groups (OR = 1.12; 95% CI, 0.59–2.10). However, pooling data for multiple mature follicle cycles showed a trend of increased pregnancy rate in the GnRH-ant group compared to the control group (OR = 1.87; 95% CI, 0.27–12.66), although the small number of studies included do not allow for any conclusions.

### PL rate

**Figure 4:**
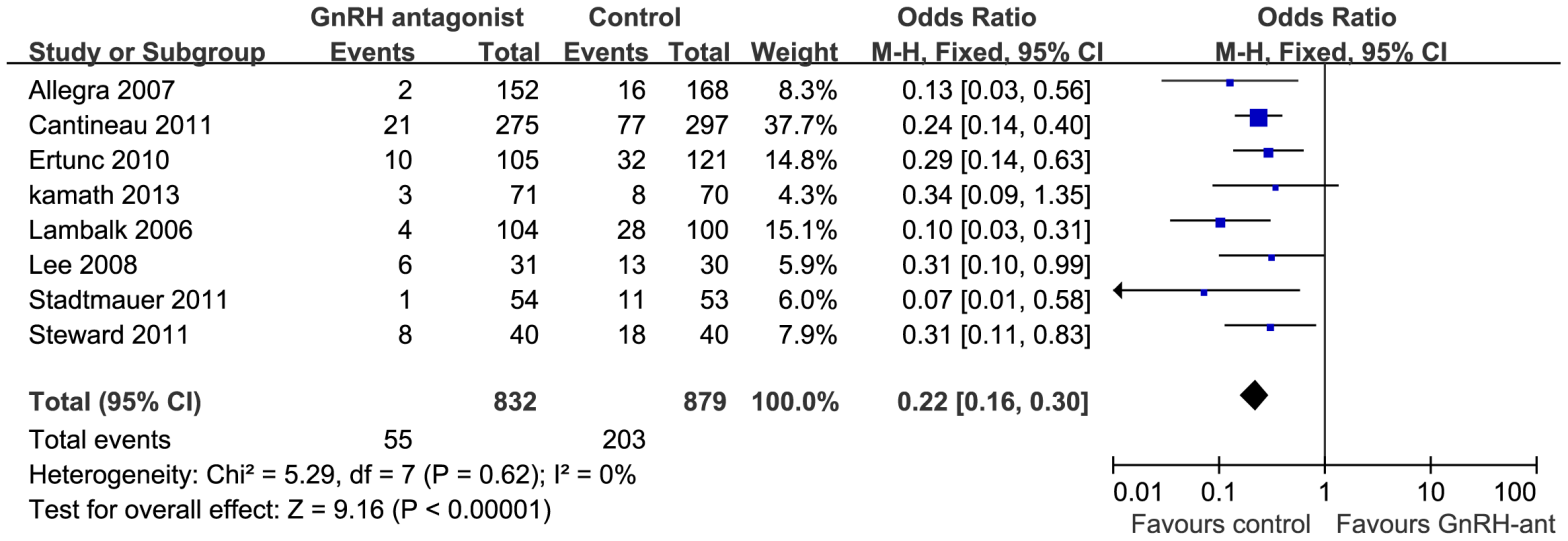
Forest plot of ORs and 95% CI of pooled trials comparing the GnRH antagonist and control for the premature luteinization rate.

Eight studies reported the PL rate [19]–[22], [25], [26], [29], [30]. The definition of PL differed slightly across the different manuscripts reviewed. Two studies [21], [29] defined PL as an increase in the LH or progesterone level on the hCG day. Six studies defined PL as an increase in the LH (≥10 IU/L) and P (≥1 ng/mL or ≥2 ng/mL) levels [19], [20], [22], [25], [26], [30]. Data pooling showed that administration of GnRH-ant in the COS/IUI cycle could significantly decrease the PL rate (OR = 0.22; 95% CI, 0.16–0.30; Figure 4). The results of the heterogeneity test were not significant (*I* ^2^ = 0% and P = 0.62), indicating that there was no statistical inconsistency among these trials.

### Other clinical outcomes

Five studies reported the OHSS rate [20], [21], [23], [24], [25]. In all cases, no OHSS occurred in any of the groups. This was due to the mild ovarian stimulation protocol and the strict cancelation policy used in all of the trials.

All studies reported the total dose of rFSH and the duration of therapy [19]–[30]. Only two studies reported an increased duration of gonadotropin administration in the GnRH- ant group compared with the control group [22], [24]. However, different initial rFSH doses and protocols were used among the various randomized clinical trials (RCTs) in this study, leading to considerably high inter-trial heterogeneity; therefore, no meta-analysis was performed on the total dose and duration of rFSH used in order to avoid extractor bias.

## Discussion

This review aimed to explore the efficacy of GnRH-ant when used as an adjuvant to COS/IUI cycles. The results of our meta-analysis showed that GnRH-ant can significantly decrease the rate of PL and cause a 42% increase in the CPR in COS/IUI treatment. Of the RCTs included in this analysis, eight included infertile women with normal ovulation, with conditions including unexplained subfertility, mild male factor infertility, or stage I–II endometriosis; two studies included women with PCOS who were infertile due to anovulation; and two studies included all the previously mentioned causes of infertility. Because ovarian hyper-response to gonadotropins and PL were especially encountered in PCOS patients, we performed subgroup analyses on the basis of the PCOS and non-PCOS populations. Results of the subgroup analysis for non-PCOS patients showed that GnRH-ant administration in COS/IUI cycles could significantly increase the pregnancy rate. This result was consistent with that of the meta-analysis performed by Kosmas et al. in 2008 [32]. However, no significant difference was found between the GnRH-ant and control groups for the PCOS patients. Nevertheless, subgroup analysis of PCOS patients was underpowered to detect differences as only two RCTs with small sample sizes were included in the review. Therefore, this result should be interpreted with caution and confirmed in future by well-designed and sufficiently powered RCTs for PCOS patients.

In this systematic review, subgroup analysis was carried out according to the mature follicle number on the hCG day. Theoretically, GnRH-ant should be more effective in improving the pregnancy rate in women with multiple follicles because such patients face a higher risk of PL. However, only two studies have reported the pregnancy rate in patients with different numbers of mature follicles on the hCG day. No significant difference was found between the GnRH-ant and control groups in single mature follicle cycles. The result for cycles with two or more mature follicles on the hCG day suggested that GnRH-ant tends to improve the clinical pregnancy outcome, although this was not statistically significant. Gomez et al. [23] reported that GnRH-ant can increase the pregnancy rate in cycles with two or more follicles ≥18 mm. However, Cantimeau et al. [20] reported no significant difference between the two groups. The different results of the two studies may be due to the different definitions of “mature follicle” in both these studies. Gomez et al. [23] defined mature follicle as follicles with diameters of ≥18 mm, but Cantimeau et al. [20] did not provide a definition of mature follicle in their subgroup analysis of multiple mature follicle cycles. Follicles with a mean diameter of 16 mm may contain immature oocytes, which tend to have a lower fertilization rate and negatively affect the pregnancy result [10]. Hence, if mature follicles are defined as follicles with diameters of ≥16 mm, it may make the difference in the CPR not obvious in subgroup analysis for cycles with ≥2 mature follicles. Therefore, well-designed RCTs with the same intervention protocol and evaluation criteria are required to draw a robust conclusion. Simultaneously, the timing of hCG and IUI administration needs to be explored in order to obtain the optimal pregnancy outcome in GnRH-ant treatment in COS/IUI cycles.

Prospective data have shown that premature LH surges occur in a significant percentage of COS cycles (22–43%), interfering with the optimal timing of the insemination or even resulting in cycle cancelation [22], [25], [26]. Premature LH has also been linked with lower pregnancy rates compared with cycles without a premature LH surge [31]. According to our meta-analysis, it seemed that the use of the GnRH-ant could decrease the incidence of PL. Our results also suggested that GnRH-ant could effectively improve the CPR by suppressing the premature LH surge and PL in COS/IUI cycles.

Two previous meta-analyses have investigated whether GnRH-ant administration significantly benefited the clinical outcome in COS/IUI programs. Kosmas et al. [32] performed a meta-analysis in 2008, which consisted of six trials with 1,069 subjects, and reported significantly higher CPR per cycle in favor of treatment with GnRH-ant. In comparison, our meta-analysis consisted of double RCTs and participants, especially including two multicenter RCTs with large sample sizes. The improvement in the quality and quantity of the studies included here strengthens the validity of the results of our meta-analysis. Furthermore, our meta-analysis included two trials that focused on patients with PCOS, and we conducted subgroup analyses for patients with and without PCOS. This work comprehensively analyzed the efficacy of GnRH-ant treatment in women with different types of infertility. Another meta-analysis performed by Cantineau et al. expressed the outcomes as live birth rates and pregnancy rates per couple, and this study revealed that GnRH-ant treatment did not improve the pregnancy rates per couple [33]. However, the number of IUI cycles administered to each couple varied from one to four. Thus, the cumulative pregnancy rate per patient with multiple IUI cycles was as high as 53.8% and obviously much higher than that in the case of single IUI cycles. Therefore, synthesizing data of pregnancy rate per couple may introduce extractor bias and interfere with the validity of the meta-analysis.

This meta-analysis has several strengths and weaknesses. It consisted of a total of 12 RCTs with 2,577 cycles. The relatively large number of included trials and participants strengthen the validity of the results of our meta-analysis. However, there was heterogeneity among the studies. The initial gonadotropin dose and ovarian simulation protocols were different across the reviewed studies, which may result in a difference in the mature follicle number, the risk of PL, and the inconsistent pregnancy rates encountered in the different studies. Furthermore, only two studies reported the use of GnRH-ant in PCOS patients undergoing COS/IUI in subgroup analysis. Therefore, the results pertaining to PCOS patients should be interpreted with caution, and more RCTs are required in the future to arrive at a more robust conclusion. Finally, it is important to highlight methodological weaknesses in existing studies, such as lack of double-blinding, and the absence of primary clinical outcome reporting (e.g. live birth rate). Such shortcomings may introduce performance and reporting bias to this review.

We concluded that GnRH-ant can effectively lower the incidence of PL and improve the CPR in patients without PCOS. In view of the scant evidence available in literature published till date, however, the role of GnRH-ant in PCOS patients undergoing COS/IUI is still unclear. In the future, well-designed RCTs, which are sufficiently powered and study different populations, should be conducted to reach useful conclusions about the use of GnRH-ant in patients undergoing COS/IUI treatment.

## Supporting Information

Checklist S1 **PRISMA Checklist.**
(DOC)
